# Bioinformatics Analysis of the Prognostic Significance of VPS16 in Hepatocellular Carcinoma and Its Role in Drug Screening

**DOI:** 10.1155/2023/2501596

**Published:** 2023-04-17

**Authors:** Xiaoming Gong, Tao Chen, Cheyu Lin, Haiqin Ping, Xin Tong, Kai Zhang, Zhaojun Chen, Caiyun Cai, Zhiyan Lu, Hengning Ke

**Affiliations:** ^1^Department of Infectious Disease, Hubei AIDS Clinical Training Center, Zhongnan Hospital of Wuhan University, Donghu Road 169, Wuchang District 430000, Wuhan, China; ^2^Department of Radiology, Xianning Central Hospital, The First Affiliated Hospital of Hubei University of Science and Technology, Xianning, Hubei, China; ^3^Department of Radiology, Zhongnan Hospital of Wuhan University, Donghu Road 169, Wuchang District 430000, Wuhan, China; ^4^Cancer Research Institute, General Hospital, Ningxia Medical University, Yinchuan, China; ^5^Wuhan Research Center for Infectious Diseases and Cancer, Chinese Academy of Medical Sciences, Wuhan, China

## Abstract

**Background:**

Vacuolar protein sorting 16 (VPS16) overexpression was recently considered related to cancer growth and drug resistance; however, little is known about whether VPS16 plays a vital role in liver hepatocellular carcinoma (LIHC).

**Methods:**

The TIMER2 online database was used to analyze the expression of VPS16 in pancancer, and the Xena Browser was used to explore the correlation between VPS16 expression level and survival time. R language was used to test the survival data of 374 LIHC cases in the TCGA database. DESeq2 was used for differentially expressed gene (DEG) analysis. The HPA database was used to verify the expression level of VPS16 in LIHC. The clusterProfiler package was used to analyze functions and related signaling pathways via GO/KEGG enrichment analysis. Drug sensitivity analysis and molecular docking technology were used to screen the most sensitive drugs targeting VPS16 molecules.

**Results:**

Pancancer analysis showed that VPS16 was highly expressed in various tumors, especially in LIHC. With the increase in the T stage and grade of LIHC, the expression level of VPS16 was also increased. The expression of VPS16 was negatively correlated with the overall survival of LIHC patients. The stage can be used as an independent prognostic factor. A total of 63 sensitive drugs were found, and 19 drugs were displaying strong molecular binding energy with VPS16.

**Conclusion:**

VPS16 may be a potential biomarker for the diagnosis and prognosis of LIHC. Drugs targeting VPS16 may be used in the treatment of LIHC in the future.

## 1. Introduction

LIHC is a malignant tumor that occurs in the liver, is the most common gastrointestinal tumor with high mortality and morbidity, and is the second leading tumor-related death—the fifth most common cancer in the world [1, 2]. Most patients are at an advanced stage at the time of diagnosis. Despite the high incidence, there are few treatment options for patients with advanced LIHC. Although ultrasound, CT, MRI, and other imaging techniques have greatly improved the diagnostic accuracy of LIHC, the high cost and limited display of microscopic lesions have also limited the early diagnosis of LIHC [3, 4]. Therefore, early diagnosis of LIHC is particularly important for timely treatment and improved prognosis. Biomarkers are easy to detect, inexpensive, noninvasive, and reproducible and thus play a significant role in LIHC [5]. Although alpha-fetoprotein (AFP) has been extensively used in routine examinations, the false-negative detection rate of early-stage microtumors is high. In addition, other lesions also have elevated AFP levels [6, 7]. Therefore, there is an urgent need to find a reliable tumor marker for LIHC. Previous studies have shown that the progression of LIHC involves multifactorial triggers and genetic mutations [8, 9]. Therefore, studies to analyze genes that are aberrantly expressed in LIHC will help to identify new therapeutic targets in the clinical setting.

Genetic research in mammals and yeast has identified four common conserved proteins (VPS11, VPS16, VPS18, and VPS33A), collectively known as the VPS-C complex [10], which is essential for the transport of large numbers of vesicles, influencing the maturation of autophagosomes [11]. Thus, the VPS-C complex plays an important regulatory role in many cellular and physiological processes, such as antigen presentation, phagocytosis of growth factor receptors, cholesterol, and lipid metabolism [12, 13]. Several reports of the VPS-C complex being associated with tumor progression also exist. Recently, Zhang et al. suggested that overexpression of VPS16 affected the progression of colon cancer and chemotherapy resistance [14]. This suggests that the VPS-C complex may be a latent target for cancer treatment. However, its expression and function in LIHC have not been investigated.

In this study, we used bioinformatics methods to analyze the expression of VPS16 on the diagnosis and prognosis of LIHC based on the TCGA database. The relevant signaling pathways, functions, and drugs targeting the VPS16 gene were analyzed and suggested a new target for the clinical diagnosis and treatment of LIHC.

## 2. Materials and Methods

### 2.1. Gene Expression Analysis of VPS16 and Differential Gene Identification of High- and Low-Expression Groups

The Human Protein Atlas (HPA) database (version: 21.1) was used to construct an mRNA expression plot for VPS16 (http://www.proteinatlas.org). To investigate VPS16 expression differences between tumor and normal tissues in different tumor types, the “Gene DE” module of Tumor Immune Estimation Resource version 2 (TIMER2) (http://timer.cistrome.org/) was used in this module. The clinical data from 374 LIHC tissues and 50 normal tissues were analyzed using R64 version 4.1.1. To get the differential expression genes between VPS16 high and low groups, DESeq2 was used to identify differentially expressed genes. We set the conditions cutting-off for |logFC| > 1 and FDR < 0.05.

### 2.2. Validation of VPS16 Protein Levels

HPA is a pathology tool that provides expression datasets for a variety of different human proteins. Therefore, we downloaded all of the VPS16 immunohistochemical data of 12 LIHC cases and 3 normal hepatocytes from the HPA database and performed immunohistochemical scores on them. Then, we compared the expression of VPS16 in LIHC and normal tissues using immunohistochemistry (IHC) (http://www.proteinatlas.org/).

### 2.3. Survival Analysis and Clinical Correlation Analysis

The median VPS16 gene expression in the samples was used as the boundary to divide VPS16 into high-expression and low-expression groups. The Kaplan-Meier survival curve analysis and the GEPIA database (http://gepia.cancer-pku.cn/) were used to analyze the correlation between VPS16 gene expression and the survival of patients with LIHC. Univariate and multifactorial Cox regression analyses were performed to explore the association between VPS16 expression and each clinical characteristic with the prognosis of patients with LIHC and to calculate the risk ratio (hazard ratio).

### 2.4. Survival Prediction

To predict the survival of a patient by column line plotting, a score for each clinical characteristic was obtained based on a scale of individual scores, and the scores for all clinical traits were added to obtain a composite score for this patient, and then the probability of one-, three-, and five-year survivals of the patient could be predicted by the column line plot constructed from the composite score.

### 2.5. GO (Gene Ontology)/KEGG (Kyoto Encyclopedia of Genes and Genomes) Enrichment Analysis

GO and KEGG pathway enrichment analysis were performed for patients between the VPS16 high-expression group and low-expression group by using the “clusterProfiler” R package, and the genes that were up- and down-regulated in the Metascape (http://metascape.org/gp/index.html) for GO and KEGG enrichment analysis, correcting for *p* value, *p* value <0.05 were statistically significant, showing the top five GO/KEGG numbers, and plotting circles and bubbles.

### 2.6. GSEA (Gene Set Enrichment Analysis)

To see which functions or pathways are active in the high or low VPS16 expression groups, we also used DESeq2 to deal with the data. The enriched graphs show the top five pathways, with a pathway at the top of the graph indicating that it is active in the high VPS16 expression group and at the bottom of the graph indicating that the pathway is active in the VPS16 low-expression group.

### 2.7. Drug Sensitivity Analysis

Nearly 75000 trials were performed on 700 tumor strains, and susceptibility information for 138 anticancer drugs was studied; the data can be found in the Genomics of Drug Sensitivity in Cancer (GDSC) project. To see which drugs had different sensitivity in the high and low VPS16 expression groups, we used Ggpubr, and ggplot2 packages as well as the drug sensitivity R package pRRophetic to obtain box plots with the horizontal coordinates representing the group of VPS16 and divided the samples into high- and low-expression groups based on gene expression; the low-expression group was represented in blue. The vertical coordinates represent drug sensitivity.

### 2.8. Core Gene Screening and Small Molecule Docking

VPS16 was used as the docking receptor. Different genes were used as ligands, AutoDockTools was used for docking, and the docking score affinity was used to screen the target with better binding activity. The docking score affinity <-5 kJ/mol^−1^ indicated that the docking molecule has docking activity, and the lower the score, the more docking activity is active. Among them, the docking score affinity <-4.25 kcal/mol^−1^ indicated that there was binding activity between the ligand and the target, and the score <-7.0 kcal/mol^−1^ indicated there was a strong docking activity between them. Protein structures were downloaded from Research Collaboratory for Structural Bioinformatics (RCSB) (http://www.rcsb.org/), Protein Data Bank (PDB), and PubChem database (https://pubchem.ncbi.nlm.nih.gov/) as “PDB” format, drug 2D structures were saved in “SDF” format, and then the Open Babel software was used to convert the “SDF” format of the drug into “PDB” format. The PyMOL software was used to remove solvent molecules and ligands and AutoDockTools 1.5.7 software was used to add hydrogen, electrons, and ROOT molecules. Preprocessing, at the same time, uses AutoDockTools 1.5.7 software for flexible molecular docking, selects the docking model with the smallest binding energy, and finally uses PyMOL software to draw the binding mode map between the target protein receptor and the drug.

### 2.9. Column Line Diagram Creation

Univariate and multivariate Cox regression models were developed to assess the association of clinical characteristics (gender, age, stage, grading, T, N, and M), and VPS16-related risk scores with overall survival (OS) in patients with LIHC and Receiver Operating Characteristic (ROC) curves were performed to assess the prognostic of the column line graph. In addition, calibration curves were used to assess the agreement between the actual and columnar line chart predicted survival probabilities.

## 3. Results

### 3.1. Gene Expression Analysis of VPS16

**Figure 1:**
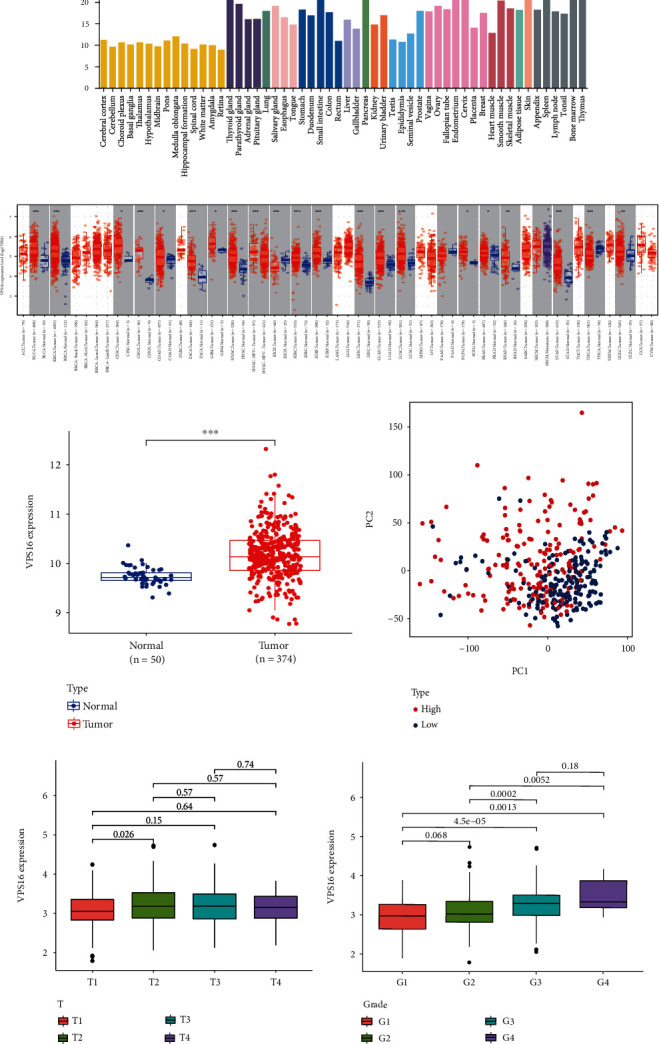
The expression of VPS16. (a) Expression levels of VSP16 mRNA in different tissues. (b) TIMER analysis identified differences in VPS16 expression between different cancer types and matched normal tissues. (c) The expression levels and differences of VPS16 in HCC tissues and normal tissues were analyzed in the TCGA database. ^∗^ represents *p* < 0.05, ^∗∗^ represents *p* < 0.01, and ^∗∗∗^ represents *p* < 0.001. (d) PCA result of VPS16 high and low groups in LIHC tissues. (e) The expression level of VPS16 in different T stages of HCC in TCGA, *p* < 0.05 indicates a statistical difference in different stages. (f) The expression level of VPS16 in different grades of HCC in TCGA, *p* < 0.05 indicates a statistical difference between different grades.

**Figure 2:**
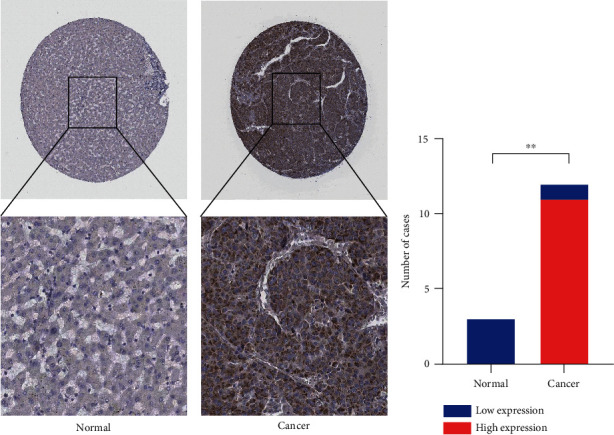
Using the HPA database to validate the protein expression of VPS16 in liver cancer tissues and normal hepatocyte tissues. (a) VPS16 IHC images of LIHC tissue and normal hepatocyte tissue in the HPA database. (b) A total of 92% (11/12, *SI* ≥ 4) of LIHC tissues were positive for VPS16 expression, while 0% (0/3, *SI* ≥ 4) of normal tissues were positive for VPS16. Data were analyzed by the Fisher exact test. ^∗^ represents *p* < 0.05, ^∗∗^ represents *p* < 0.01, and ^∗∗∗^ represents *p* < 0.001.

The HPA, Genotype-Tissue Expression (GTEx) project, and (function annotation of the mammalian) FANTOM5 datasets revealed that VPS16 is highly expressed in the thyroid gland and spleen, and it is also enriched in the pancreas and bone marrow (Figure 1(a)). What is more, single-cell RNA-seq revealed that VPS16 is highly expressed in proximal enterocytes and NK cells (Supplementary Figure S1). Compared with the normal tissue distribution of mRNA, VPS16 showed low specificity. Its mRNA expression was remarkably increased in various tumors including UCEC, STAD, READ, PRAD, PCPG, LUSC, LUAD, LIHC, KIRP, KIRC, HNSC-HPV, GBM, ESCA, COAD, CHOL, CESC, and BLCA. Downregulated VPS16 was observed in KICH (Figure 1(b)). To verify the expression of VPS16 in LIHC, we analyzed the mRNA levels of 374 LIHC tumor samples and 50 normal samples in TCGA and found that VPS16 was highly expressed in LIHC (Figure 1(c)). In addition, PCA visualization analysis was performed on the VPS16 high- and low-expression group samples in the LIHC samples, and the result revealed that the VPS16 high- and low-expression group samples had significantly distinguished transcriptional profiles (Figure 1(d)). To further validate VPS16 protein expression, we searched immunohistochemistry (IHC) staining information from the HPA. The clinical characteristics and VPS16 *H*-score of 12 cases of liver cancer and 3 cases of normal hepatocytes in the HPA database were analyzed. The 12 cases of liver cancer were 52-76 years old, with an average age of 65.4 years old. Males accounted for 7/12 (Table 1). The IHC results revealed an obvious abundance of VPS16 protein in LIHC tissues (Supplementary Figure S2). The protein expression of VPS16 in LIHC was significantly higher than that in normal hepatocyte tissues (*p* < 0.01) (Figures 2(a) and 2(b), and the above findings showed that VPS16 may promote carcinogenesis progression in various tumor types, especially in LIHC.

### 3.2. Correlation between VPS16 Expression and Prognosis of LIHC

**Figure 3:**
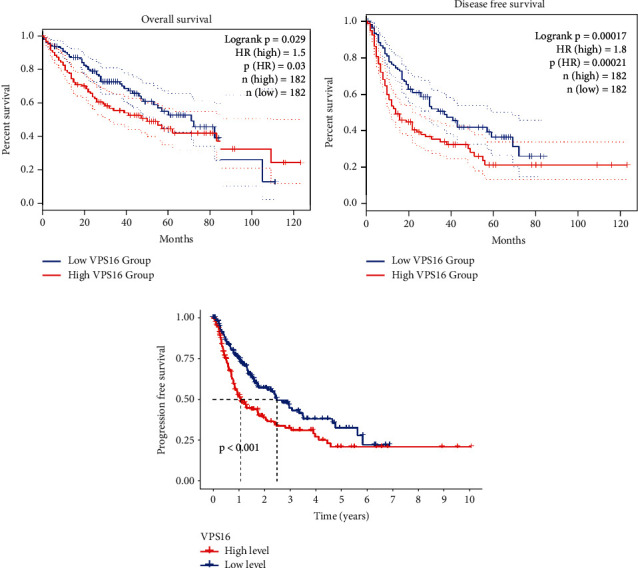
Kaplan-Meier survival curve of hepatic carcinoma with high and low VPS16 expression analyzed by the GEPAI 2 database and validation from the TCGA database. (a) High VPS16 expression was related to worse OS in LIHC (*n* = 364). (b) High VPS16 expression was related to worse DFS in LIHC (*n* = 364). (c) High VPS16 expression was related to worse PFS in LIHC (*n* = 374) from TCGA.

To further study the potential prognostic value of VPS16, we next investigated the correlation between the VPS16 expression and the prognosis of patients with LIHC by using GEPIA2. We found that higher VPS16 expression was negatively correlated with OS in the case of LIHC (*p* = 0.03) (Figure 3(a)), and higher VPS16 expression was also correlated with shorter progression-free survival (FDS) in LIHC (*p* = 2.1 × 10^−4^) (Figure 3(b)). Furthermore, we also confirmed that VPS16 was negatively correlated with FDS in 374 LIHC samples from the TCGA database (Figure 3(c)) (*p* < 0.001).

### 3.3. VPS16-Related Signaling Pathways

**Figure 4:**
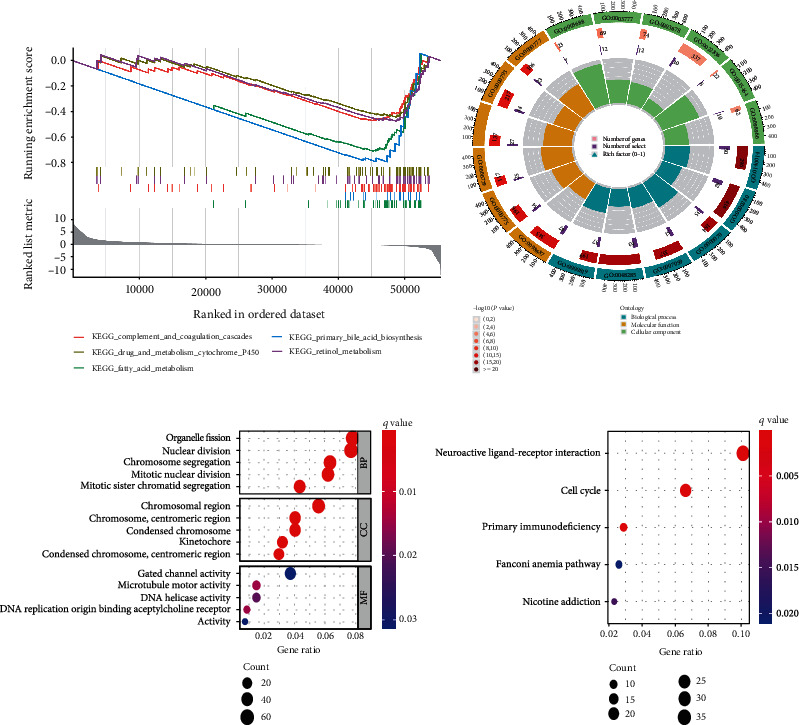
GSEA, GO, and KEGG analysis. (a) GSEA analysis showed that five pathways were most enriched in the VPS16 high-expression group. (b) The circle diagram visualizes biological processes, molecular function, and cellular component enrichment. (c, d) GO and KEGG enrichment analysis of VPS16-derived genes.

Using GSEA, we further analyzed the signaling pathways involved in VPS16, the high-expression group of VPS16 was enriched in several metabolic pathways including coagulation cascade, drug metabolism, cytochrome P450, and fatty acid metabolism. These signaling pathways are closely correlated with primary bile acid biosynthesis and retinol metabolism (Figure 4(a)). Next, we explored the possible biological functions of the VPS16 protein. Enrichment analysis of GO and KEGG has shown the top five of each (Figures 4(b) and 4(c)). The signaling pathways involved in VPS16 include neuroactive ligand-receptor interaction, cell cycle, primary immunodeficiency, Fanconi anemia pathway, and nicotine addiction (Figure 4(d)).

### 3.4. Targeted Drug Sensitivity Analysis and Molecular Docking

**Figure 5:**
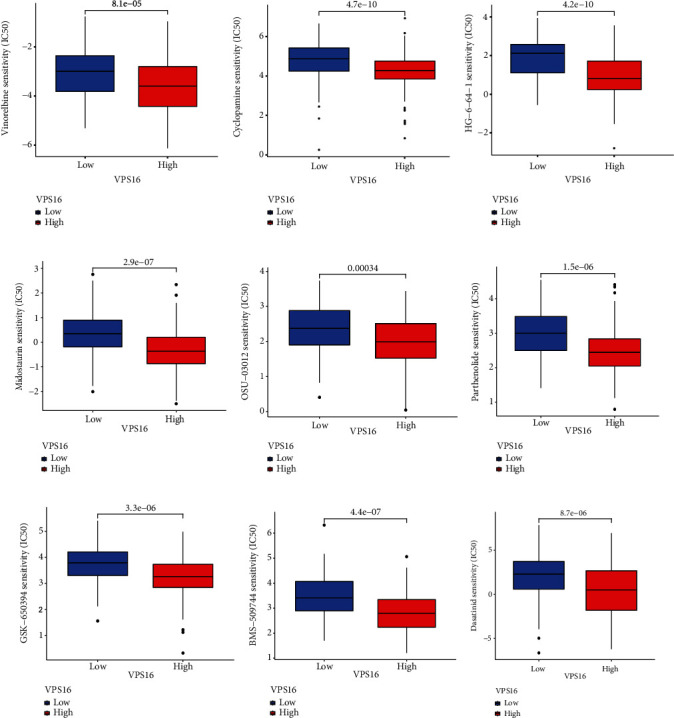
Some of the results showed significant differences in drug sensitivity analysis between high and low VPS16 expression groups. (a–i) Drug-sensitive box plot of the top nine drugs for molecular docking.

**Figure 6:**
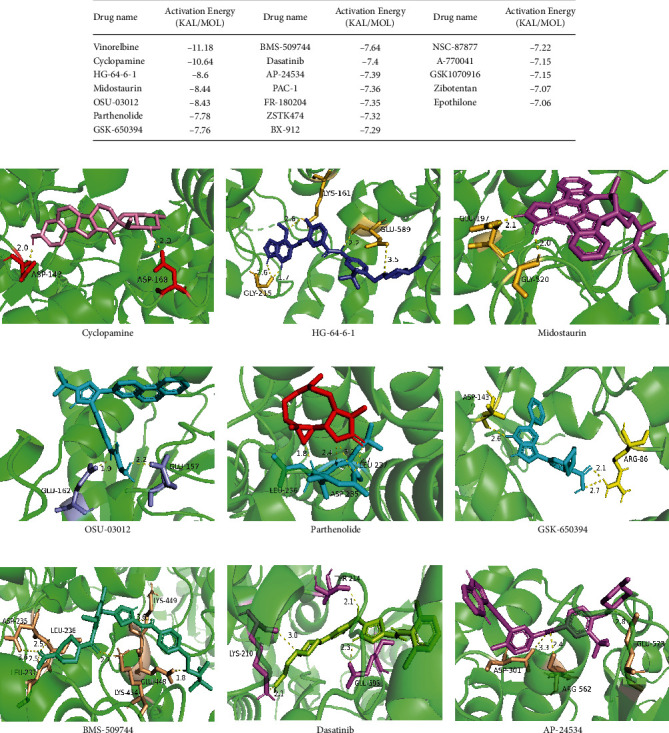
Molecular docking diagram of VPS16 with sensitive drugs. (a) Drug name with a binding activation energy of sensitive drugs less than -7. (b–j) Molecular docking diagram of VPS16 and the top nine sensitive drugs.

To understand the therapeutic effects of drugs targeting the VPS16 target in LIHC. Using drug sensitivity R packet analysis, we identified a total of 63 targeted drugs that showed lower IC_50_ values in the VPS16 high-expression group of LIHC, as shown in Supplementary Table 1. These nine drugs had the lowest activation energy to bind to VPS16 and all showed lower half maximal inhibitory concentration (IC_50_) values in the high VPS16 expression group, with *p* value <0.001 (Figures 5(a)–5(i)). The order of binding activity was vinorelbine, cyclopamine, HG-6-64-1, midostaurin, OSU-03012, parthenolide, GSK-650394, BMS-509744, dasatinib, and AP-24534; the activation energy was shown in Figure 6(a). Finally, we also established a reliable line graph to predict the prognosis of LIHC. This indicates that these targeted drugs were more sensitive to the treatment of LIHC in the VPS16 high-expression group. The docking diagram and binding energy of the drug and VPS16 molecule were shown in Figures 6(b)–6(j).

### 3.5. Establishing a Reliable Line Chart to Predict the Prognosis of LIHC

**Figure 7:**
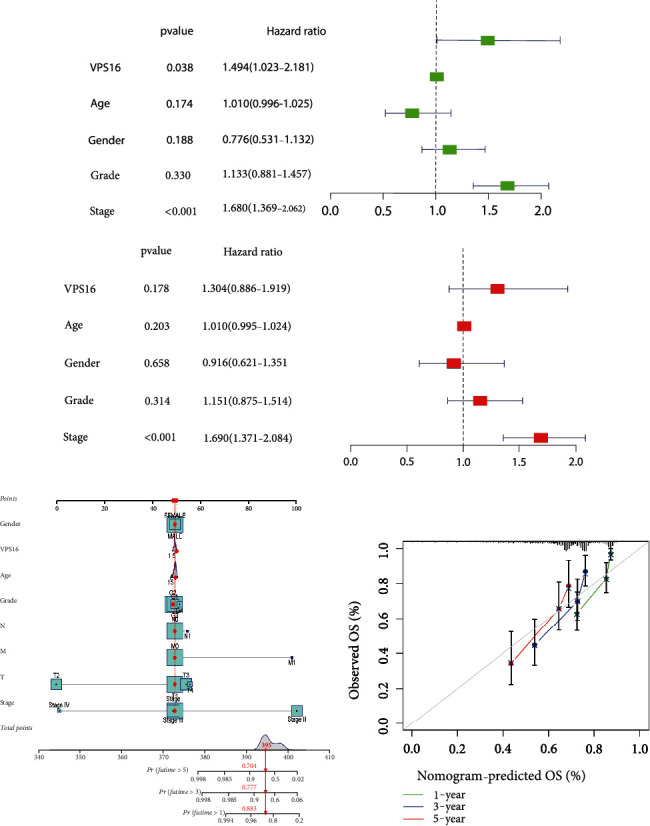
Establishment of a reliable nomogram for prediction of LIHC prognosis. (a, b) Uni- and multivariate Cox regression models are conducted to uncover the association between clinical features and VPS16-derived risk score. (c) A prognostic nomogram is exploited by integrating independent prognostic indicators (gender, VPS16, age, grade, N, M, T, and stage) to estimate one-, three-, and five-year survival probabilities. (d) The calibration plots show the predicted one-, three-, and five-year OS versus actually associated survival time.

Univariate Cox regression analysis revealed significant associations between gender, age, stage, grade, T, N, M, and VPS16-derived risk scores and prognosis of LIHC (Figure 7(a)). Among them, the stage could be used as an independent prognostic indicator for LIHC (Figure 7(b)). By integrating the above clinical traits, this study estimated the survival outcome of patients with LIHC using the column line graph (Figure 7(c)), and the ROC curve confirmed the ideal outcome of patient survival (Supplementary Figure S3). In addition, we also evaluated the performance of the predicted column line graph with calibration curves, and our data demonstrated that the 1-, 3-, and 5-year survival times predicted by the column line graph were close to the actual survival time (Figure 7(d)). These data indicated that the column line graph has strong predictive power.

## 4. Discussion

LIHC is on the top list of causes of cancer-related deaths worldwide [15]. Because of its aggressive nature, ease of metastasis, and frequent recurrence, LIHC has been a major health problem endangering global health [16]. Although some progress has been made in the treatment of LIHC, the prognosis of patients with LIHC is still less than satisfactory [17]. The occurrence and progression of LIHC involve an accumulation of genetic factors and epigenetic alterations [18]. Therefore, it is particularly important to investigate the aberrantly expressed genes and potential mechanisms in the development and progression of LIHC and to screen for drugs that are sensitive to them to explore new therapeutic targets for LIHC.

In this study, we analyzed the expression of VPS16 in tumor tissues and normal tissues by LIHC using the TCGA database. The results showed that VPS16 expression was significantly increased in various tumors, especially in LIHC. It was elevated in patients with a high clinical stage as well as high pathological grade compared to those with lower grades. The IHC results from the HPA database revealed an obvious abundance of VPS16 protein level in LIHC tissues. Meanwhile, the protein expression of VPS16 in LIHC was significantly higher than that in normal hepatocyte tissues. This also further validated that VPS16 was differentially expressed in LIHC and normal hepatocyte tissues and has the potential as a tumor marker in the future.

In addition, GSEA analysis showed that the five pathways enriched in the high-expression group of VPS16 include complement and coagulation cascades, drug metabolism, cytochrome P450, fatty acid metabolism, primary bile acid biosynthesis, and retinol metabolism. These pathways suggest that the abnormal expression of VPS16 may affect normal liver metabolism. GO and KEGG enrichment analysis were performed to screen the differentially expressed genes in the high-expression group and the low-expression group of VPS16. The results showed that the upregulated genes in the high-expression group of VPS16 were mainly concentrated in the mitotic cell cycle and DNA replication pathways, suggesting that VPS16 may play a role in promoting cancer by affecting the cell cycle and mitosis. Meanwhile, GSEA, GO, and KEGG analysis showed that increased expression of VPS16 caused abnormal fatty acid metabolism, bile acid synthesis, and retinol metabolism, suggesting that VPS16 may also be related to hepatic metabolic pathways.

To better identify VPS16 interaction potential genes, we used the GeneMANIA software to generate a visual interactive network; we found that the strongest five genes correlated with VPS16 were VIPAS39, VPS33A, VPS18, VPS11, and VPS41. Among them, VPS11 and VPS18 are also one of the core components of VPS-C. Some recent studies have shown that the VPS-C protein is a promising target for cancer therapy [19, 20], suggesting that VPS16 and related genes may have similar effects. It also lays a theoretical foundation for VPS16 to be a potential diagnostic and prognostic target of LIHC.

Taking VPS16 as the target, we divided LIHC samples into the VPS16 high-expression group and the VPS16 low-expression group. We screened 63 drugs sensitive to the VPS16 high-expression group. These drugs had lower IC_50_ values compared with the VPS16 low-expression group, indicating that these drugs were more sensitive to the treatment of LIHC in the VPS16 high-expression group. Then, we performed molecular docking on these drugs and the VPS16 protein, respectively. The molecular docking results showed that a total of 51 sensitive drugs spontaneously bound to the core target protein VPS16, and there were 19 drugs with strong molecular binding energy and binding energy less than -7 [21], among which the nine drugs with the strongest binding to VPS16 were ranked according to the binding energy; they were vinorelbine, cyclopamine, HG-6-64-1, midostaurin, OSU-03012, parthenolide, GSK-650394, BMS-509744, and dasatinib. The higher the binding energy, the closer the drug binds to VPS16. Vinorelbine is an antimitotic agent that inhibits the proliferation of tumor cells [22–24]. It has been widely used in a variety of tumor clinical trials, such as breast cancer, lung cancer, lymphoma, advanced solid tumors, malignant mesothelioma, head and neck neoplasms, colon cancer, prostate cancer, cervical adenocarcinoma, low-grade glioma, melanoma (skin), rhabdomyosarcoma, sarcoma, and ovarian cancer. Cyclopamine is an antagonist of the Hedgehog pathway and a selective inhibitor of smo. Tumor reduction can be induced in a genetic mouse model of myeloma by Hh inhibitors that bind to smo, such as HhAntag and naturally occurring cyclopamine [25]. Cyclopamine can cause long-term tumor disappearance of xenogeneic cells. Tumors disappeared within 12 days after treatment with cyclopamine [26]. HG6-64-1 is a highly potent and selective B-Raf inhibitor, derived from patent WO2011090738A2, example 9 (XI-1); Ba/F3 cells that transformed with B-rafV600E have an IC_50_ of 0.09 *μ*M. Midostaurin is an orally active, reversible multitargeted protein kinase inhibitor such as PKC*α*/*β*/*γ*, Syk, Flk-1, Akt, PKA, c-Kit, c-Fgr, c-Src, FLT3, and PDFR*β* [27, 28]. Midostaurin also upregulates endothelial nitric oxide synthase (eNOS) gene expression and shows powerful anticancer effects [29]. In addition, it has a strong antiproliferative ability against various tumors and normal cell lines in vitro and can reverse pgp-mediated multidrug resistance [27]. The results showed that OSU-03012 (AR-12) had a good inhibitory effect on PDK-1. It can inhibit PI3K/Akt/mTOR signaling pathway. Parthenolide is a sesquiterpene lactone found in the traditional Chinese medicine parthenolide. It had a certain anti-inflammatory effect on the activation of NF-*κ*B; HDAC1 protein was also inhibited. Parthenolide has a certain effect on the growth of NSCLC cells [30]. GSK650394, a novel SGK inhibitor, inhibits insulin-induced phosphorylation of PKB-Ser473 at 3 *μ*M but has no effect on the phosphorylation of PRAS40-Ser246 in hormone-deprived cells or prevents insulin-induced phosphorylation of PKB-Ser473 phosphorylation [31, 32]. BMS-509744 is a highly potent and selective ATP inhibitor. It also significantly reduces ovalbumin-induced pneumonia in allergic/asthmatic mice [33]. Dasatinib is a potent ATP-competitive, orally active dual Src/Bcr-Abl inhibitor. Dasatinib has a certain promotion effect on cell apoptosis and autophagy [34]. The results of these drugs' in vitro or in vivo tests or clinical trials showed that they had a certain inhibitory effect on some tumors. Although some drugs have been used in many tumors, they have not been used in clinical trials and studies of LIHC so far, and there were no reports about the role of VPS16 in these drugs. We also confirmed the predicted tight binding of these sensitive drugs to VPS16 through molecular docking which provided a theoretical basis for the clinical application of these drugs in LIHC in the future.

In this study, we also developed a reliable nomogram to predict the prognosis of LIHC, using univariate and multivariate Cox regression to analyze the significant association of clinical traits (sex, age, grade, stage, T, N, and M) and VPS16-derived risk scores with LIHC prognosis. Among them, the stage can be used as an independent prognostic indicator of liver cancer. We also evaluated the performance of predicting the nomogram with calibration curves and demonstrated that the nomogram's one-, three-, and five-year survival times were close to the actual survival time.

The shortcomings of this study are as follows: (1) the mRNA and protein levels of VPS16 show differences between LIHC and normal tissues, which may have adverse effects on prognosis. However, further studies are needed to investigate the expression of this marker in blood and body fluids in the future. (2) Although some targeted drugs targeting VPS16 have been screened to provide a theoretical basis for the early treatment of LIHC in the future, the efficacy and safety of these drugs still need to be further and repeatedly verified in large sample experiments and clinical trials in the future.

In conclusion, we found that VPS16 was significantly upregulated in LIHC for the first time, and it upregulates with the increase of grade and stage. The expression of VPS16 was negatively correlated with the prognosis of LIHC. We also screened 19 sensitive drugs which could be strongly combined with VPS16, and they may be a good choice for LIHC treatment in the future. To better serve the clinical practice in the future, we also established a relatively stable model to predict the prognosis of patients.

## Figures and Tables

**Table 1:** The clinical characteristics and protein expression of VPS16 in LIHC and normal tissues.

Number	Gender	Age (y)	Intensity	Quantity	SI	Staining
Cancer 1	F	73	3	4	12	High
Cancer 2	M	67	2	4	12	High
Cancer 3	M	59	2	4	8	High
Cancer 4	F	73	2	4	8	High
Cancer 5	F	58	1	1	1	Low
Cancer 6	F	53	3	4	12	High
Cancer 7	M	76	2	4	8	High
Cancer 8	M	67	3	4	12	High
Cancer 9	M	70	2	4	8	High
Cancer 10	M	72	2	4	8	High
Cancer 11	F	52	2	4	8	High
Cancer 12	M	65	2	3	6	High
Normal 1	F	54	2	1	2	Low
Normal 2	F	63	2	1	2	Low
Normal 3	M	55	2	1	2	Low

The data was downloaded from the HPA database (https://www.proteinatlas.org/). VPS16 expression intensity (0, 1+, 2+, and 3+) and positive cell percentage were evaluated, which were 0 (0%), 1 (1~25%), 2 (25~50%), 3 (50~75%), and 4 (75~100%), respectively. The staining index (SI) was calculated as follows: SI = (strength score in 1) × (positive staining score in 2), SI ≤ 3 was considered the low expression, and SI ≥ 4 was considered the high expression.

## Data Availability

The data used to support the findings of this study are available from the corresponding author upon request.

## Supplementary Materials

Supplementary 1 Supplementary Table 1: sensitive drugs that showed lower IC_50_ values in the VPS16 high-expression group of LIHC.

Supplementary 2 Supplementary Figure S1: single-cell RNA-seq of VPS16. Supplementary Figure S2: immunohistochemical images of 12 cases of liver cancer and 3 cases of normal hepatocytes cells from the HPA database. Supplementary Figure S3: the ROC curve.

Supplementary 3 Supplementary data. Supplementary package R.
