# Supplementary material for: Bioinformatics Analysis of the Prognostic Significance of VPS16 in Hepatocellular Carcinoma and Its Role in Drug Screening
Source: Biomed Res Int. 2023 Apr 17;2023:2501596. doi: 10.1155/2023/2501596 (PMC10137196; doi:10.1155/2023/2501596)
Supplement: Supplementary 1 — Supplementary Table 1: sensitive drugs that showed lower IC50 values in the VPS16 high-expression group of LIHC. [file 2501596.f1.docx]

**Supplement Table1.** Sensitive drugs that showed lower IC50 values in the VPS16 high expression group of LIHC

| Sensitive drug name | Targeting gene |
| --- | --- |
| 5-Fluorouracil | VPS16 |
| A-443654 | VPS16 |
| A-770041 | VPS16 |
| AKT inhibitor VIII | VPS16 |
| AP-24534 | VPS16 |
| AS601245 | VPS16 |
| AS605240 | VPS16 |
| AT-7519 | VPS16 |
| BAY 61-3606 | VPS16 |
| BI-2536 | VPS16 |
| BMS345541 | VPS16 |
| BMS-509744 | VPS16 |
| BX-912 | VPS16 |
| CAL-101 | VPS16 |
| CMK | VPS16 |
| Cyclopamine | VPS16 |
| Dasatinib | VPS16 |
| Epothilone B | VPS16 |
| FMK | VPS16 |
| FR-180204 | VPS16 |
| Gemcitabine | VPS16 |
| Genentech Cpd 10 | VPS16 |
| GSK-650394 | VPS16 |
| GSK1070916 | VPS16 |
| GSK1904529A | VPS16 |
| GW843682X | VPS16 |
| HG-6-64-1 | VPS16 |
| IPA-3 | VPS16 |
| Ispinesib Mesylate | VPS16 |
| JNK-9L | VPS16 |
| JW-7-52-1 | VPS16 |
| KIN001-102 | VPS16 |
| LFM-A13 | VPS16 |
| MG-132 | VPS16 |
| Midostaurin | VPS16 |
| NPK76-II-72-1 | VPS16 |
| NSC-87877 | VPS16 |
| Obatoclax Mesylate | VPS16 |
| OSU-03012 | VPS16 |
| PAC-1 | VPS16 |
| Paclitaxel | VPS16 |
| Parthenolide | VPS16 |
| PF-562271 | VPS16 |
| QS11 | VPS16 |
| Rapamycin | VPS16 |
| Ruxolitinib | VPS16 |
| Shikonin | VPS16 |
| STF-62247 | VPS16 |
| S-Trityl-L-cysteine | VPS16 |
| Sunitinib | VPS16 |
| TAE684 | VPS16 |
| Thapsigargin | VPS16 |
| Tipifarnib | VPS16 |
| Vinorelbine | VPS16 |
| VX-11e | VPS16 |
| VX-680 | VPS16 |
| WH-4-023 | VPS16 |
| WZ-1-84 | VPS16 |
| XL-184 | VPS16 |
| XMD8-85 | VPS16 |
| XMD14-99 | VPS16 |
| Zibotentan | VPS16 |
| Z-LLNle-CHO | VPS16 |
| ZSTK474 | VPS16 |
