# Supplementary material for: Bioinformatics Analysis of the Prognostic Significance of VPS16 in Hepatocellular Carcinoma and Its Role in Drug Screening
Source: Biomed Res Int. 2023 Apr 17;2023:2501596. doi: 10.1155/2023/2501596 (PMC10137196; doi:10.1155/2023/2501596)
Supplement: Supplementary 2 — Supplementary Figure S1: single-cell RNA-seq of VPS16. Supplementary Figure S2: immunohistochemical images of 12 cases of liver cancer and 3 cases of normal hepatocytes cells from the HPA database. Supplementary Figure S3: the ROC curve. [file 2501596.f2.docx]

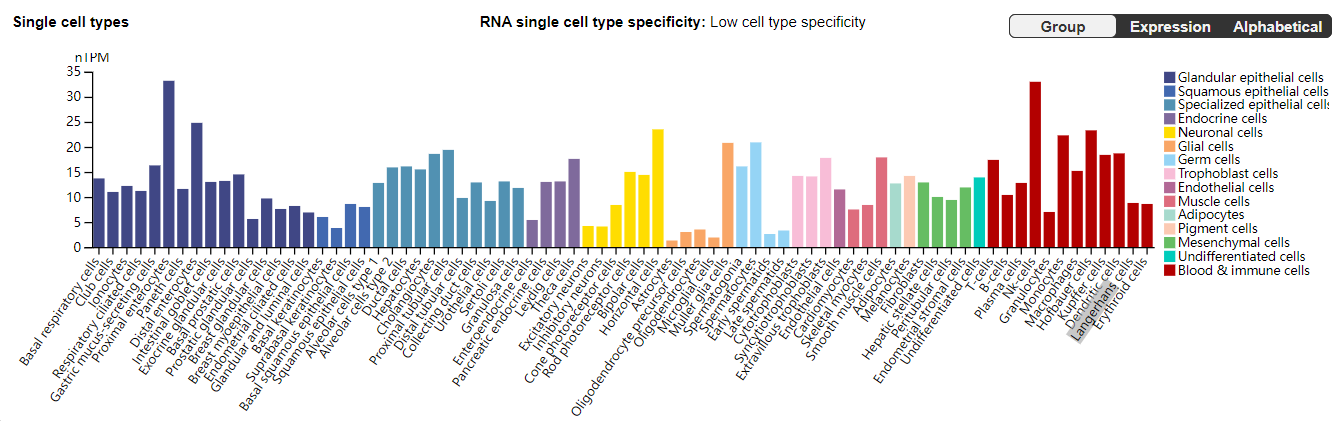


**Supplementary Figure S1**. single-cell RNA-seq of VPS16.


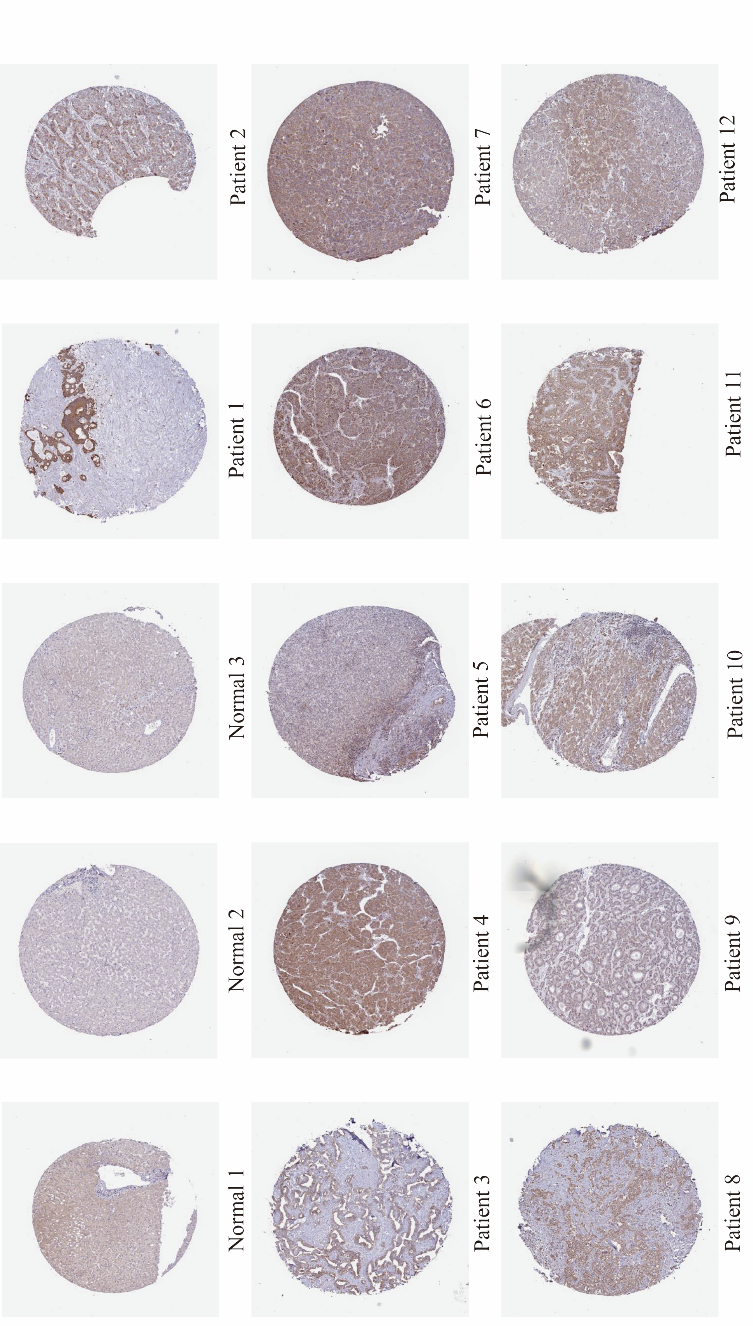


**Supplementary Figure S2**. Immunohistochemical images of 12 cases of liver cancer and 3 cases of normal hepatocytes cells from the HPA database. Antibody product name: HPA048661.


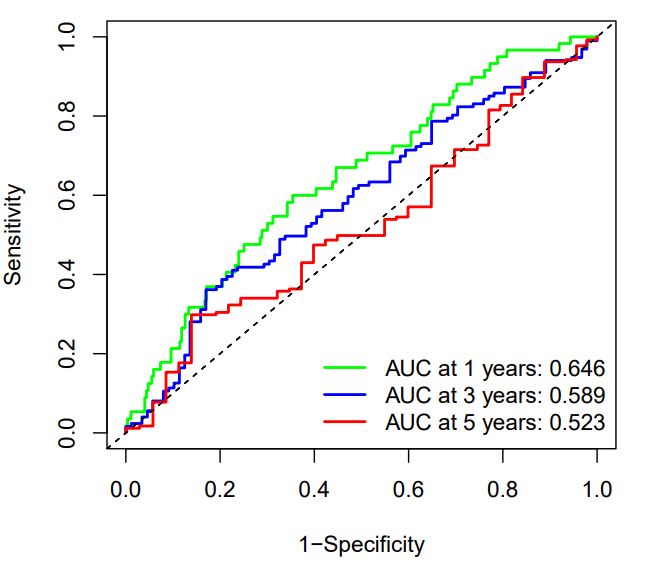


**Supplementary Figure S2**. the ROC Curve.

**Abbreviations:**

AFP: alpha-fetoprotein,VPS16: Vacuolar Protein Sorting16, BLCA: Bladder Urothelial Carcinoma, CESC: Cervical squamous cell carcinoma and endocercial adenocarcinoma, CHOL: Cholangio carcinoma, COAD: Colon adenocarcinoma, ESCA: Esophageal carcinoma, GBM: Glioblastoma multiforme, HNSC: Head and Neck squamous cell carcinoma, KIRC: Kidney renal clear cell carcinoma, KIRP: Kidney renal papillary cell carcinoma, LIHC: Liver hepatocellular carcinoma, LUAD: Lung squamous cell carcinoma, LUSC: Lung squamous cell carcinoma, PCPG: Pheochromocytoma and Paraganglioma, PRAD: Prostate adenocarcinoma, READ: Rectum adenocarcinoma, STAD: Stomach adenocarcinoma, UCEC: Uterine Corpus Endometrial Carcinoma, HPA: The Human Protein Atlas, TIMER2:Tumor Immune Estimate Resource version 2 ,GO: Gene Ontology, KEGG: Kyoto Encyclopedia of Genes and Genomes, GEPIA: Gene Expression Profiling Interactive Analysis, OS: Overall Survival, FDS: progression-free survival, ROC: Receiver Operating Characteristic Curve, GTEx: Genotype-Tissue Expression Project, GSEA: Gene Set Enrichment Analysis, RCSB: Research Collaboratory for Structural Bioinformatics, PDB: Protein Data Bank, IHC: immunohistochemistry, IC50: half maximal inhibitory concentration, SI: staining index
